# Correction: Excitons in metal halide perovskite nanoplatelets: an effective mass description of polaronic, dielectric and quantum confinement effects

**DOI:** 10.1039/d4na90041c

**Published:** 2024-04-03

**Authors:** Jose L. Movilla, Josep Planelles, Juan I. Climente

**Affiliations:** a Departament d’Educació i Didàctiques Específiques, Universitat Jaume I Av. Sos Baynat, s/n 12071 Castelló Spain; b Departament de Química Física i Analítica, Universitat Jaume I Av. Sos Baynat, s/n 12071 Castelló Spain climente@uji.es

## Abstract

Correction for ‘Excitons in metal halide perovskite nanoplatelets: an effective mass description of polaronic, dielectric and quantum confinement effects’ by Jose L. Movilla *et al.*, *Nanoscale Adv.*, 2023, **5**, 6093–6101, https://doi.org/10.1039/d3na00592e.

The authors regret a data post-processing error relating to the calculation of the binding energies shown in panels (c) and (d) of **Fig. 2** in the original paper. The correct panels are displayed here in Fig. 1. Contrary to what is stated in the paper, Fig. 1(d) shows that the Haken potential provides quantitative estimates of experimental binding energies, without the need to resort to phenomenological corrections such as the Bajaj potential. In fact, the Bajaj potential underestimates the exciton binding energy in few-layer structures. Otherwise, the qualitative analysis of the figure is unaffected, as well as the computational codes provided along with the original article.

**Fig. 1:**
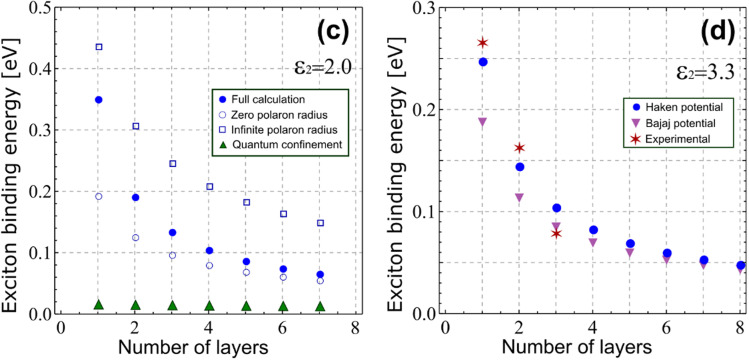
Corrected panels (c) and (d) of **Fig. 2** in the original paper. (c) Exciton binding energy. The organic barriers have *ε*_2_ = 2. Results are depicted for different degrees of approximation in the model. Blue dots: full calculation. Empty circles: zero polaron radius (*V*_Y_ = 0, *ε*_s1_ = 22). Squares: infinite polaron radius (*V*_Y_ = 0, *ε*_s1_ = 5.6). Green triangles: quantum confinement only (*V*_Y_ = 0, *ε*_2_ = *ε*_s1_ = 22). (d) Exciton binding energy for organic barriers with *ε*_2_ = 3.3. Blue dots: full calculation with Haken potential. Purple triangles: full calculation with Bajaj potential. Stars: experimental data for (PEA)_2_(MA)_*n*−1_Pb_*n*_I_3*n*+1_, from *J. Phys. Chem. Lett.*, 2021, **12**, 1638–1643.

The Royal Society of Chemistry apologises for these errors and any consequent inconvenience to authors and readers.

## Supplementary Material

